# European *EHBP1L1* Genotyping Survey of Dyserythropoietic Anemia and Myopathy Syndrome in English Springer Spaniels

**DOI:** 10.3390/vetsci11120596

**Published:** 2024-11-26

**Authors:** Sarah Østergård Jensen, Alexandra Kehl, Urs Giger

**Affiliations:** 1 AniCura Small Animal Referral Hospital Bagarmossen, Ljusnevägen 17, Bagarmossen, SE-12848 Stockholm, Sweden; 2 LABOKLIN GmbH & Co. KG, Steubenstr. 4, DE-97688 Bad Kissingen, Germany; alexandra.kehl@laboklin.com; 3 Department of Small Animal Internal Medicine, Vetsuisse Faculty, University of Zürich, Winterthurerstrasse 260, CH-8057 Zürich, Switzerland; giger@vet.upenn.edu

**Keywords:** canine, centronuclear myopathy, hereditary, iron deficiency, metarubricytosis, microcytosis, prevalence, real-time PCR

## Abstract

Dyserythropoietic anemia and myopathy syndrome (DAMS) with neonatal losses was recently characterized as an autosomal recessive disorder caused by an *EHBP1L1* frameshift variant in English Springer Spaniels (ESSPs). The frequency and dissemination of the mutation remained unknown. The EHBP1L1 protein is essential for muscle function, and the Rab8/10-EHBP1L1-Bin1-dynamin axis participates in nuclear polarization during the enucleation of erythroblasts. Lack of EHBP1L1 function decreases enucleation, leading to increased numbers of nucleated erythrocytes, which are characteristic of DAMS. A genotyping survey for the *EHBP1L1* variant was conducted based upon submitted samples of ESSPs from Europe. DNA was extracted, and a real-time PCR assay, with allele-specific TaqMan probes for *EHBP1L1* wild-type and frameshift deletion, was applied. Between September 2022 and August 2024, 803 samples were received from 18 European countries. The *EHBP1L1* mutant allele frequency was 9.7%, including 4 homozygous dogs and 148 heterozygotes. The mutant *EHBP1L1* allele was found in 13 countries. A total of 6 homozygous and 73 heterozygous ESSPs reported on an open database could be tracked to an original common ancestor. Although the survey is biased, it indicates that the mutant *EHBP1L1* variant is disseminated in the breed and across Europe. The genotyping of ESSPs is recommended to diagnose DAMS and guide breeders.

## Simple Summary

A syndromic hereditary disorder known as dyserythropoietic anemia and myopathy syndrome (DAMS) with perinatal losses was recently described, from its clinical signs to the discovered gene identification and mutation, in English Springer Spaniel dogs from Sweden and Australia. The disorder is characterized by defective red blood and muscle cells, leading to anemia and muscle wasting, with affected dogs having progressive illness including difficulty moving, drinking, and eating. This is an autosomal recessive trait, and two copies of the mutant gene (allele) from both parent dogs are needed. The presence and distribution of the gene mutation in Europe was unknown. Thus, the aim of this study was to survey European countries to assess the mutant allele prevalence. Samples were sent to one veterinary laboratory in Germany for genotyping, and the frequency of the mutation was calculated. Results from 803 dogs were available, and the frequency of the mutant allele was a staggering 9.7%. Although the survey is biased, this is surprisingly high since DAMS was only recently characterized and genotyping was voluntary. Centralized, official breeding schemes with obligatory genotyping and transparency of results would be beneficial for planned optimal breeding to avoid the spread of the mutant allele, puppy mortality, and the production of affected dogs.

## 1. Introduction

Hereditary disorders are common in dogs, with many being described from clinical signs to causative gene defects, with some serving as disease models. Both hereditary myopathies and erythrocytic defects have been investigated and described in depth for several different entities in dogs [1,2,3,4,5,6]. A few studies have reported pleiotropic defects involving erythrocytes and myocytes such as in phosphofructokinase deficiency and dyserythropoietic anemia and myopathy syndrome (DAMS) in show ESSPs [4,5,6,7]. A family of ESSPs with microcytic anemia, inappropriate metarubricytosis, and myopathy from Sweden, related to ESSPs from Australia (clinically described three decades earlier), was recently documented to be homozygous for a single base deletion in the *EHBP1L1* gene, leading to a deleterious frameshift [4,5].

Recent ongoing research on the function of EHBP1L1 has shown that this protein is important for cell polarity and particularly for the enucleation of erythroblasts, erythrocyte membrane stability, and nuclear positioning in skeletal myocytes. These are all cells that are affected in dogs which are homozygous for the deletion and frameshift *EHBP1L1* mutation and are thereby suffering from DAMS [4,8]. In a study of *EHBP1L1^−/−^* mice produced in the laboratory, the mice all died within a day of birth, which complements our previous findings in dogs, in which perinatal death was also seen in individuals homozygous for the mutant allele [4,8]. The function of EHBP1L1 in epithelial cells also involves cell polarization, where Rab8 and Bin1 are responsible for the regulation of apical transport, meaning that mice lacking the protein had truncated and sparse microvilli in the small intestine [9].

The prevalence of the pathogenic *EHBP1L1* variant in the ESSP breed and its distribution in Europe have been unknown. However, based upon our studies, commercial genetic testing has been developed and is now available in Europe [10,11] and under development in the US [12].

The aim of this study was to investigate the frequency of the causative *EHBP1L1* variant in the ESSP population in Europe. When available, pedigrees from affected dogs and carriers were reviewed for the presence of the common ancestor as previously described [4,5].

## 2. Materials and Methods

Cheek swab or blood samples from ESSPs in Europe were received for *EHBP1L1* genotyping at a major veterinary diagnostic laboratory (Labogen, Laboklin GmBH & Co. KG., Bad Kissingen, Germany) between September 2022 and August 2024. Since all samples were received for medical and diagnostic purposes, no institutional or official ethical approvals were required.

Genomic DNA was isolated from ethylenediaminetetraacetic acid (EDTA) blood samples or buccal swabs using MagNAPure (Roche, Basel, Switzerland) with the MagNA Pure DNA and viral NA kit according to the manufacturer’s instructions. A TaqMan assay with allele-specific TaqMan probes for *EHBP1L1* wild-type and frameshift deletion was applied for genotyping using LC480 (Roche, Basel, Switzerland).

The dog owners and/or veterinary clinicians of heterozygous and homozygous dogs were offered contact with one of the authors (SØJ) for guidance regarding breeding of the heterozygous dogs, and for guidance on palliative treatment for the homozygous dogs, providing care and a temporarily improved quality of life. Furthermore, the ancestry the previously identified common ancestor of DAMS in heterozygous dogs which was identified in our last study [4], and the results of DAMS testing openly posted on Fleckenbase [13], were examined.

## 3. Results

The study population consisted of 803 ESSPs from 18 European countries. Of these, 464 were females, 314 were males, and 25 dogs were of unknown sex. Most dogs were from Finland, Germany, Sweden, and the United Kingdom (Table 1), with only a few dogs genotyped in other countries. Four dogs were found to be homozygous for the *EHBP1L1* variant (two males and two females), and they were from Austria, Germany, and Sweden (Table 1). One hundred and forty-eight dogs were heterozygous for the *EHBP1L1* mutant allele without any known clinical disease. They were from 13 countries across Europe (Table 1). Albeit this is not a randomized survey, and it is likely biased by breeder interests, the frequency of the mutant *EHBP1L1* allele was 9.7% among European ESSPs in this study, which is unexpectedly high for an, until recently, unknown hereditary disorder or clinical syndrome in dogs and other species.

On the open registry of Fleckenbase, owners and breeders of ESSPs had reported results from 73 heterozygous and 6 homozygous dogs, genotyped for the *EHBP1L1* variant [13].

## 4. Discussion

While the first ESSP dogs with dyserythropoietic anemia and myopathy syndrome (DAMS) and neonatal losses were reported in Scandinavia just a couple of years ago [4], the herein-reported *EHBP1L1* mutant allele frequency among European ESSPs appears surprisingly high. Indeed, a couple of ESSPs showing clinical signs of DAMS were already described in Australia more than three decades ago, thus predicting a more worldwide distribution [5]. All the clinically affected ESSP dogs were confirmed to be homozygous for the frameshift deletion in *EHBP1L1* reported in our previous study, in which pedigree analyses revealed a common ancestry to the genotyped ESSPs from Sweden [4,5]. In the current study, for those homozygous and heterozygous dogs for which identity and genotyping results were posted on Fleckenbase, the ancestry of 6 homozygous affected dogs and all 73 ESSPs genotyped as heterozygous could be tracked to the previously identified common ancestor [4,13].

Based upon the apparently high *EHBP1L1* mutant allele frequency from the genotyping survey in this study, more than four homozygous affected dogs might have been expected. However, this gene defect in ESSP dogs, as well as in mice and humans, is associated with perinatal deaths [4,8,14]. These losses could have been accepted by breeders as part of the common canine puppy mortality complex, and/or breeders choose not to genotype and report possible homozygous perinatal deaths. Also, owners and breeders with dogs showing classical clinical syndromic signs of DAMS might not have chosen to confirm the DAMS diagnosis by genotyping. For dogs born prior to the discovery of the causative *EHBP1L1* variant, they might well have been euthanized due to anemia and myopathy owing to a lack of effective therapy and poor quality of life.

The results of this survey confirm that the *EHBP1L1* mutant allele is present in several European countries, but there is a vast difference in the number of dogs tested in each country (Table 1). It is therefore possible that the *EHBP1L1* mutation is also present in other countries and continents that, as of now, have not been screened or only have dogs carrying wild-type alleles. To that end, a heterozygous dog has been reported in the US, confirming the presence of the mutant allele there [13]. It is also possible that breeders with dogs genotyped heterozygous for the *EHBP1L1* variant chose not to test the littermates, parents, or offspring of these dogs to avoid the risk of having many heterozygous and clinically unaffected dogs, which can make the planning of breeding more complicated. Thus, there is also a risk that the frequency of heterozygous dogs is higher than what was observed in this study.

In the UK, genotyping has been part of an official breeding scheme for registered ESSPs since fall 2023 [15], whereas results from tested dogs in the rest of Europe are reported on a voluntary basis on either breed club websites and/or online databases, such as the German webpage for spaniels Fleckenbase.de [13]. The implementation of official breeding schemes, with obligatory genotyping and registration of results of intended breeding dogs in different European kennel clubs, would increase the amount of information about the prevalence and dissemination of the mutant allele in more European countries, eliminate the risk of owners and breeders not reporting results, and thereby avoid the spread of the mutant allele and the production of affected ESSPs.

## 5. Conclusions

In conclusion, this study shows that the frameshift mutation in the *EHBP1L1* gene, leading to DAMS and perinatal losses in homozygous dogs, is widely spread among the European population of ESSPs. Most recent information on the function of the EHBP1L1 protein sheds light on the pathophysiologic mechanism behind the clinical, clinicopathological, and histopathological features seen in homozygous dogs and adds to the knowledge of the disease in the relatively few DAMS-affected dogs that have been described so far. All ESSPs genotyped as homozygous and heterozygous, which were publicly identified, had the previously reported common ancestor in their pedigree [13]. Centralized, kennel club-organized breeding schemes with obligatory genotyping and transparency in the reporting of results are imperative for informed planned breeding of ESSPs to avoid possible resorption in utero with subsequent small litter sizes, perinatal deaths, and the production of homozygous individuals affected by DAMS.

## Figures and Tables

**Table 1:** *EHBP1L1* genotyping survey of English Springer Spaniel dogs in Europe. Numbers in bold refer to dogs heterozygous or homozygous for the *EHBP1L1* variant.

European Country	*EHBP1L1* Genotype			Total
	Wild Type/Normal	Heterozygous	Homozygous	
Austria	23	**7**	**1**	31
Bulgaria	2	0	0	2
Czech Republic	33	**6**	0	39
Croatia	4	**3**	0	7
Denmark	13	**1**	0	14
Estonia	3	0	0	3
Finland	84	**20**	0	104
Germany	128	**34**	**2**	164
Hungary	2	0	0	2
Netherlands	8	0	0	8
Norway	33	**4**	0	37
Poland	51	**7**	0	58
Romania	0	**1**	0	1
Sweden	97	**16**	**1**	114
Switzerland	7	**2**	0	9
Spain	7	**1**	0	8
Ukraine	1	0	0	1
United Kingdom	154	**47**	0	201
Total	651	148	**4**	803

## Data Availability

The datasets used and/or analyzed during the current study are included in the manuscript.
